# Mental simulation and compulsive buying: a multiple mediation model through impulse buying and self-control

**DOI:** 10.3389/fpsyg.2025.1507031

**Published:** 2025-03-17

**Authors:** Xiaowei Duan

**Affiliations:** School of Economics and Management, Zhejiang Shuren University, Hangzhou, China

**Keywords:** impulse buying, compulsive buying, self-control, mental simulation, process simulation, outcome simulation

## Abstract

This study explores the multiple mediation effects of impulse buying and a self-control failure on the relationship between two types of mental simulation—outcome and process simulation—and compulsive buying. We collected 202 responses using a web-based survey, which were used as a final example. The respondents for this study were recruited through the web-based survey platform Nown Survey. Using structural equation modeling and PROCESS for SPSS (Model 6), we estimated the internal consistency of measurements and tested the established hypotheses. The main findings confirm the distinct impacts of the two types of mental simulation on primary constructs and the multiple mediation effects of impulse buying and self-control failure on the associations between the two types of mental simulation and compulsive buying. Despite the multiple mediating effects of impulse buying and self-control failure between the two types of mental simulation and compulsive buying, process simulation is positively associated with both impulse buying and compulsive buying, while outcome simulation is significantly related only to impulse buying. Our results supplement existing literature by applying new insights into the relationships between mental simulation and compulsive buying. Further, our findings may help marketers to establish strategies based on the divergent roles of the two types of mental simulation to motivate consumers’ purchase behaviors. Finally, the limitations and directions for future research are discussed.

## 1 Introduction

Compulsive buying is characterized by addictive and excessive buying behaviors, which are associated with uncontrollable impulses to purchase something. These actions are almost always accompanied by negative emotional responses such as anxiety, frustration, and depression (Brook et al., 2015; Ridgway et al., 2008). Existing compulsive buying literature shows that more than 5.8% of adults in the United States has declared a tendency toward compulsive purchases (Japutra et al., 2025). Moreover, an empirical study on compulsive buyers in a range of countries reports that an estimated 3.4–6.9% of consumers are compulsive buyers; this rate is especially high among young adults, ranging from 2 to 16% (Maraz et al., 2016). However, some research still confounds compulsive buying and impulse buying, and even uses these two terms interchangeably in academic settings (Kellett and Bolton, 2009). There is a clear distinction between repetitive, excessive buying behavior (i.e., compulsive buying) and unplanned, immediate purchase behavior (i.e., impulse buying; Darrat et al., 2016; Zafar et al., 2021). The concept of impulse buying places greater emphasis on a predisposition to purchase spontaneously and immediately, exhibits a diminished regard for presumable outcomes, and is often accompanied by positive feelings (Amos et al., 2014; Sharma et al., 2010). In contrast, compulsive buying exhibits elements of both impulsive (i.e., a frequent loss of impulse control over buying) and obsessive traits (i.e., a repetitive and addictive purchase behavior), which are associated with negative emotional states (Japutra et al., 2025).

Existing compulsive buying literature has identified a plethora of serious consequences of compulsive buying. For instance, some findings reveal that compulsive buying behaviors may lead to both a decrease in autonomy and structural financial difficulties (Koran et al., 2006). Likewise, compulsive buying is remarkably correlated with damaged interpersonal complications and a stream of negative emotional responses, such as guilt, psychological distress, and higher depression rates (Gallagher et al., 2017; O’Guinn and Faber, 1989). Compulsive buying is also considered to be an impaired function in aligning consumers’ behaviors with their perceptions of wishes or expectations from unanimous others (Brook et al., 2015). However, only a limited amount of research has explored the drivers of consumers’ compulsive buying behaviors. A typical example of this is that consumers’ feelings of anxiety stemming from impulse buying are highly linked to their compulsive purchase behaviors (Darrat et al., 2016). It is, therefore, clear that extant studies on compulsive buying are still largely silent on antecedents to compulsive buying, and that more conceivable triggers of compulsive buying should be investigated.

This study aims to investigate the relationship between mental simulation and compulsive buying, with a focus on impulse buying and self-control failure as mediators within a serial mediation model. The motivation behind this research stems from the increasing prevalence of compulsive buying behaviors in consumer culture, which can lead to significant psychological and financial consequences (Gallagher et al., 2017; Japutra et al., 2025). Mental simulation, which can be further divided into outcome simulation and process simulation, plays a crucial role in shaping consumers’ decisions. Outcome simulation helps individuals envision the potential benefits of their purchases (Zhou et al., 2024), while process simulation influences the way they approach shopping experiences (Zhao et al., 2011). This duality may lead to heightened impulsivity and diminished self-control, ultimately contributing to compulsive buying tendencies. By exploring these dynamics, this research seeks to provide valuable insights into how mental simulation can inform strategies for mitigating compulsive buying behaviors.

In line with this statement, we propose the research objectives of this study. First, we explore the distinctive effects of two types of mental simulation (i.e., outcome and process simulation) on impulse buying, compulsive buying, and self-control failure. Second, we advance both conceptualizations and operationalizations of impulse buying and compulsive buying to identify the distinction and association between these two constructs. Third, we investigate the multiple mediation effects of impulse buying and self-control failure on the relationship between the two types of mental simulation and compulsive buying. The results suggest a significantly positive association between process simulation and compulsive buying is confirmed, whereas the link between outcome simulation and compulsive buying is statistically insignificant. In terms of mediation effects, the serial multiple mediation effects of impulse buying and self-control failure on the relationships between the two types of mental simulation and compulsive buying are empirically supported. The remainder of this article is organized as follows: we start by reviewing the existing literature and developing a research model; we then describe the hypotheses testing results and relevant statistics in the next section; and finally, the implications and limitations are presented.

## 2 Literature review and hypothesis development

### 2.1 Mental simulation

Mental simulation is the cognitive construction of hypothetical events that involves anticipation of desirable outcomes and how one can bring them about (Zhou et al., 2024; Riskind and Calvete, 2020; Taylor et al., 1998). The concept of mental simulation has been explored in both the psychology and marketing sectors. For instance, advertisers usually aim to engage consumers in a narrative process to imagine an interaction with the product (Zhao et al., 2011), because envisioning an interaction with the product can make the products seem more “real” or favorable (Taylor et al., 1998). Furthermore, mental simulation can help consumers to establish a positive evaluation of and a favorable attitude toward simulated products which, in turn, directly impacts their behavioral intentions (Escalas, 2004). Informed by existing literature on the topic, mental simulation has been conceptually dichotomized into two distinct types: process simulation, which focuses on the process of reaching a desirable end state, and outcome simulation, which focuses on the end state the individual wants to achieve (Escalas and Luce, 2004).

Considering these two types, outcome simulation encourages greater focus on the importance of desirability (i.e., the pursuit of desirable outcomes) and the reasons for using a product, whereas process simulation places greater emphasis on its feasibility (i.e., the possibility of achieving desirable outcomes) and how to cope with presumable constraints (Castaño et al., 2008; Thompson et al., 2009). In this perspective, existing empirical evidence signifies that the two types of mental simulation are not equally effective in influencing consumers’ decision-making. Process simulation has been shown to be conducive to bolstering duration framing effects (Ülkümen and Thomas, 2013), directing focus to near-future events or objects (Zhao et al., 2011), motivating goal achievement (Pham and Taylor, 1999), and promoting the persuasiveness of advertisements (Escalas and Luce, 2004). In contrast, outcome simulation is likely to direct focus to distant-future events or objects (Zhao et al., 2011), lower consumers’ performance uncertainty, and stimulate positive feelings (Castaño et al., 2008).

### 2.2 Mental simulation and compulsive buying

In the traditional academic setting, individuals’ compulsive buying behaviors are often interpreted solely as problematic purchasing behavior or obsessive-compulsive disorder by numerous researchers (Miltenberger et al., 2003; O’Guinn and Faber, 1989). As the body of research on compulsive buying grows, an increasing number of researchers are investigating the supplementary behavioral or psychological features of compulsive buyers, rather than encouraging a greater focus on the individual’s repetitive and excessive purchasing behavior itself (Darrat et al., 2016; Gallagher et al., 2017; Mueller et al., 2011). That is, compulsive buyers are more prone to exhibit lower self-esteem and higher levels of fantasy, compared with normal individuals (O’Guinn and Faber, 1989); this is usually accompanied by the embodiment of vulnerability and a sense of impulse control failure over buying (Kukar-Kinney et al., 2016).

The existing literature on compulsive buying has empirically determined a plethora of presumable antecedents to and consequences of compulsive buying. For instance, Dittmar (2005) confirms the strong associations among individuals’ compulsive buying behaviors, self-discrepancies, and ideal-self buying motivations. In a similar vein, the findings of Mueller et al. (2011) report that compulsive buying is remarkably related to both materialistic orientations and negative mood states (e.g., anxiety, loneliness, stressfulness, and depression). Furthermore, the conceivable outcomes resulting from individuals’ compulsive buying behaviors incorporate the subjective perception of self-control failure, damaged domestic relationships (O’Guinn and Faber, 1989), financial dilemmas, interpersonal complications (Gallagher et al., 2017), feelings of guilt (McQueen et al., 2014), and psychological distress (Brook et al., 2015).

Seen in this light, individuals who engage in compulsive behaviors appear to cope with negative affect aroused by obsessions—such as anxiety, frustration, and depression—while, in turn, temporarily relieving the unpleasant state or situation through the buying process itself (Brook et al., 2015; Gallagher et al., 2017; O’Guinn and Faber, 1989). The primary motivation for compulsive buying is to compensate for negative emotional states and to achieve psychological gratification through ritualistic buying behavior, rather than to garner utility from the possession of purchased commodities (Koran et al., 2006; O’Guinn and Faber, 1989). Informed by the aforementioned reports, this study defines compulsive buying as a repetitive, excessive, and purposeful purchase behavior with a tendency to display a chronic loss of impulse control over buying, which may lead to substantial financial and interpersonal difficulties (Japutra et al., 2019; McQueen et al., 2014; Ridgway et al., 2008).

Previous studies also show that compulsive buying is most often accompanied by a stream of analytic and deliberative cognitive components, including inappropriate beliefs about purchased objects and deliberations in the decision-making processes (Kyrios et al., 2004). As noted earlier, effective mental simulation contributes to promoting a positive attitude toward a product and a favorable brand evaluation; this, in turn, boosts consumers’ confidence in the actual purchase behaviors (Yim et al., 2021). Nonetheless, two divergent types of mental simulation play distinctive roles in consumers’ decision-making processes. Process simulation places an emphasis on cognitive domains such as plans for reaching the end state, whereas outcome simulation encourages greater focus on affective domains such as positive feelings of goal achievement (Pham and Taylor, 1999). In this regard, it is anticipated that two types of mental simulation exert differentiated impacts on consumers’ compulsive buying behaviors. That is, the consumer’s outcome simulation is detrimental to the cognitive components of their compulsive buying behaviors; this, in turn, leads to a negative compulsive buying behavior. Differing from outcome simulation, process simulation has an antithetical impact on compulsive buying; in other words, the individual’s process simulation is conducive to evoking a compulsive buying behavior. Accordingly, the following hypotheses were formulated:

*H1a*: Outcome simulation relates negatively to compulsive buying.

*H1b*: Process simulation relates positively to compulsive buying.

### 2.3 The mediating role of impulse buying

The term impulse buying, sometimes called impulsive purchases, is described as an unplanned purchase behavior by many existing studies (Iyer, 1989; Rook and Gardner, 1993; Stern, 1962). A typical example is Stern (1962), who conceptually identifies four types of impulse buying from the perspective of unplanned purchase behaviors: (1) pure impulse buying (purchase behavior with a complete absence of pre-purchase planning); (2) suggested impulse buying (may occur based on a strong in-store recommendation or temptation); (3) reminded impulse buying (may occur when standing in front of the shelf and recalling a previously existed buying demand); (4) planned impulse buying (characterized by a partial presence of pre-purchase planning, for instance, a price range for the product has been decided before entering a brick-and-mortar store).

With the development of researchers’ understanding of impulse buying behaviors, an increasing number of empirical studies are pointing out the significant distinction between impulse buying behavior and generic unplanned purchase behavior (Amos et al., 2014; Zhang et al., 2010). Impulse buying behavior can be viewed as a kind of unplanned purchase behaviors in a broad sense; however, it is inappropriate to classify wholly unplanned purchase behaviors as impulsive purchase behaviors (Zhang et al., 2010). More specifically, compared to generic unplanned purchase behaviors, impulse buying is positively associated with a sudden and irresistible urge to buy (Amos et al., 2014) and is primarily driven by positive feelings such as delight, joyfulness, and excitement (Kathuria and Bakshi, 2024). By and large, the most recent studies on impulse buying behaviors are typically centered around internal psychological processes rooted in impulsive purchase behaviors, rather than external shopping list behaviors (Rook and Gardner, 1993).

Consistent with the aforementioned statements, generic unplanned purchase behaviors and impulsive purchase behaviors differ in several aspects. First, consumers with buying impulsiveness tend to experience an automatic, abrupt, and forceful urge to purchase something (Rook and Fisher, 1995). Second, for the most part, impulse buying behavior is accompanied by high-arousal emotions, such as enjoyment, cheerfulness, and pleasure (Verplanken et al., 2005). Third, impulsive purchases usually preclude any wise, careful, and thoughtful consideration of presumable costs or consequences (Sharma et al., 2010). Fourth, impulse buyers are prone to placing greater value on short-term gratification and a strong incentive value, rather than deferring the purchase to do comparison shopping or seek useful advice from peers (Jones et al., 2003). Lastly, impulsive purchasing is associated with both undermined utilitarian considerations and temporal self-control failure in a general sense (Rook and Fisher, 1995; Verplanken et al., 2005). As suggested above, impulse buying is defined as a sudden, spontaneous, unreflective, and hedonic purchase behavior with a lack of deliberate contemplation of alternative implications or presumable consequences (Hofmann et al., 2009; Sharma et al., 2010; Li et al., 2024).

Impulse buying is typically centered around affective states, such as high-arousal feelings (e.g., excitement, pleasure) and an irresistible urge to purchase something (Rook, 1987). Moreover, impulse buying affect has been disaggregated into two dimensions: dispositional and situational factors (Beatty and Ferrell, 1998). Dispositional factors involve with impulse buying tendency (i.e., the extent to which an individual is likely to purchase something spontaneously, immoderately, and thoughtlessly; Jones et al., 2003) and shopping enjoyment tendency (i.e., the degree to which an individual experiences greater enjoyment or pleasure in a shopping behavior than generic others; Kim and Kim, 2008). Situational factors include the display arrangement of products, the appearance of products, store environments (e.g., music, smell), and in-store promotions, among others (Beatty and Ferrell, 1998). Furthermore, as previously noted, outcome simulation is highly related to affects; in contrast, process simulation is remarkably correlated to cognition (Pham and Taylor, 1999). Therefore, it can be posited that outcome simulation is effective in simulating the affective components of impulse buying, and motivating impulse buying behavior. However, process simulation exerts a contradictory effect on impulse buying; this means that process simulation is negatively associated with impulse buying.

When we consider compulsive buying, the bulk of existing research relevant to compulsive buying manifests that individuals’ impulse buying behaviors are significantly related to their compulsive buying behaviors (Brook et al., 2015; Faber et al., 1995; Flight et al., 2012). Ridgway et al. (2008) claim that compulsive buying displays components of both the impulsive trait (i.e., the irresistible urge to purchase something) and obsessive disorder (i.e., repetitive and excessive purchase behavior). An empirical study by Darrat et al. (2016) indicates that consumers’ impulse buying is likely to fuel negative mood states (e.g., anxiety), which can, in turn, lead to compulsive buying behavior. Moreover, another work reports that compulsive buying is remarkably associated with three aspects of individuals’ impulsiveness: urgency, lack of perseverance, and lack of premeditation (Billieux et al., 2008). Furthermore, the mediating role of impulse buying has been confirmed by numerous researchers. For example, a study by Lo et al. (2016) reports that consumers’ motivation factors are conducive to promoting their online impulse buying; this, in turn, leads to a sense of self-control failure. Relatedly, extant research findings reveal that impulse buying positively mediates the link between materialism and compulsive buying (Israel and Jena, 2018). Accordingly, we hypothesize the mediating role of impulse buying between mental simulation and compulsive buying, and propose the following hypotheses:

*H2a*: Impulse buying mediates the link between outcome simulation and compulsive buying.

*H2b*: Impulse buying mediates the link between process simulation and compulsive buying.

### 2.4 The mediating role of self-control

Individuals may experience a situation in which they are torn between the urge to seek immediate gratification and a call to behave reasonably (Hofmann et al., 2009). This means that the pivotal role of individuals’ self-control is to alleviate the incompatibility between their long-term goals and their urge toward immediate hedonic fulfillment (Hofmann et al., 2009). Hoch and Loewenstein (1991) interpret consumers’ self-control as an ever-changing conflict between the desire for short-term hedonic satisfaction and the willpower to refrain from behaving irrationally. For instance, most individuals may know that the fruit salad is a rational alternative for dessert, compared to chocolate cake; yet, they still prefer the chocolate cake because of a desire for strong hedonic temptations of immediate gratification (Hofmann et al., 2009). Moreover, Averill’s (1973) findings reveal that individuals’ self-control comprises the ability to interpret and manage unwanted information and the ability to align their behaviors with what they believe. Against this backdrop, self-control is defined as an individual’s capacity to alter or override their states or responses, such as thoughts, emotions, performance, and behavioral tendencies (Baumeister, 2002; Gailliot et al., 2007).

Based on the foregoing assertions, the two types of mental simulation are likely to influence consumers’ impulse and compulsive buying behaviors through various underlying mechanisms. Drawing from this logic, we propose that the two types of mental simulation play differential roles in influencing consumers’ abilities to control their purchase behaviors. Parallel to extant studies, activated process simulation is positively associated with individuals’ goal achievement, performance in buying behaviors, and self-regulation (Taylor et al., 1998). Likewise, process simulation is effective in facilitating emotional regulation to mitigate feelings of anxiety, lessening affective uncertainty and switching costs, and increasing the likelihood of problem solving (Castaño et al., 2008; Taylor et al., 1998). In contrast, outcome simulation is conducive to decreasing performance uncertainty and fueling strong positive feelings (Castaño et al., 2008). Similarly, outcome simulation is likely to diminish impulse control over buying, which, in turn, leads to a loss of self-control (Escalas, 2004). Hence, this study supposes that process simulation is positively associated with self-control failure, whereas outcome simulation is negatively associated with self-control failure.

In line with existing studies on compulsive buying, a shortfall of self-control and difficult-to-control urges to purchase commodities are considered as the predominant drivers of compulsive buying behavior (Achtziger et al., 2015; Darrat et al., 2016; Horváth et al., 2015). Referring to Claes et al. (2011), individuals who purchase compulsively tend to display lower levels of self-control and embrace an inhibitory self-control failure, an activation self-control failure, and an attentional self-control failure. The relationship between self-control and compulsive buying is scrutinized through two underlying mechanisms. First, as a behavioral addiction problem, compulsive buying is usually accompanied by extensive self-regulatory problems (e.g., a lack of impulse control over shopping), which is detrimental to personal concerns and interpersonal facilitation (Grant et al., 2010; Rose, 2007). Second, compulsive buyers are more likely to experience self-regulatory difficulties and exhibit higher levels of self-control resource depletion. These individuals typically tend to perceive a strong negative emotional arousal and a sense of self-control failure; they then, in turn, abandon themselves to the situation in which they might purchase excessively (i.e., compulsive buying) when at a higher level of self-control resource depletion (Rose, 2007; Vohs and Faber, 2007). Drawing from this logic, the current study posits that an individual’s diminished ability for self-control may lead to ritualistic compulsive buying behavior.

Eexisting literature also confirms the mediating role of self-control in a wide variety of academic settings. For instance, Roberts and Manolis (2012) identify three critical antecedents (i.e., ego depletion, goal conflicts, and self-monitoring) to self-control and confirm the negative association between self-control and impulse buying. In doing so, the mediating role of self-control among these three factors and impulse buying was also statistically confirmed. Informed by the above review, the current study presumes the mediating role of self-control failure between mental simulation and compulsive buying, and advances the following hypotheses:

*H3a*: Self-control failure mediates the link between outcome simulation and compulsive buying.

*H3b*: Self-control failure mediates the link between process simulation and compulsive buying.

### 2.5 Multiple mediation effects of impulse buying and self-control

Exploring the relationship between impulse buying behavior and self-control failure has been a crucial theme underlying many recent studies on impulse buying (Baumeister, 2002; Hofmann et al., 2008; Roberts and Manolis, 2012; Sultan et al., 2012). In line with extant studies on self-control, Baumeister (2002) identifies three decisive causes of self-control failure, and asserts that if any of these elements ceases to function, it may lead to loss of self-control. First, a conspicuous contradiction between immediate gratification and one’s long-term goals may erode one’s self-control ability; second, a failure to surveil one’s own behavior may cause serious damage to one’s self-control; and third, a self-control resource (i.e., cognitive capacity) depletion may render self-control difficult. In the setting of impulse buying, a tremendous range of existing studies illuminate the negative association between impulse buying and self-control (Roberts and Manolis, 2012; Sultan et al., 2012; Vohs and Faber, 2007). Sultan et al. (2012) suggest that an individual’s self-control ability is remarkably correlated with lower levels of impulse buying (e.g., deliberate considerations of presumable outcomes and contemplations of future consequences). Relatedly, individuals with high levels of control are more likely to weigh alternatives carefully and make a rational decision, whereas individuals low in control are prone to show a diminished regard for presumable costs or outcomes (Roberts and Manolis, 2012). More specifically, individuals indulge themselves with impulsive purchases to garner immediate hedonic satisfaction; yet, they may feel out-of-control after impulse buying (Baumeister, 2002; Lo et al., 2016). Given the above discussion, the current study postulates that individuals’ impulsive purchase behaviors may make their self-control ability less effective.

**Figure 1:**
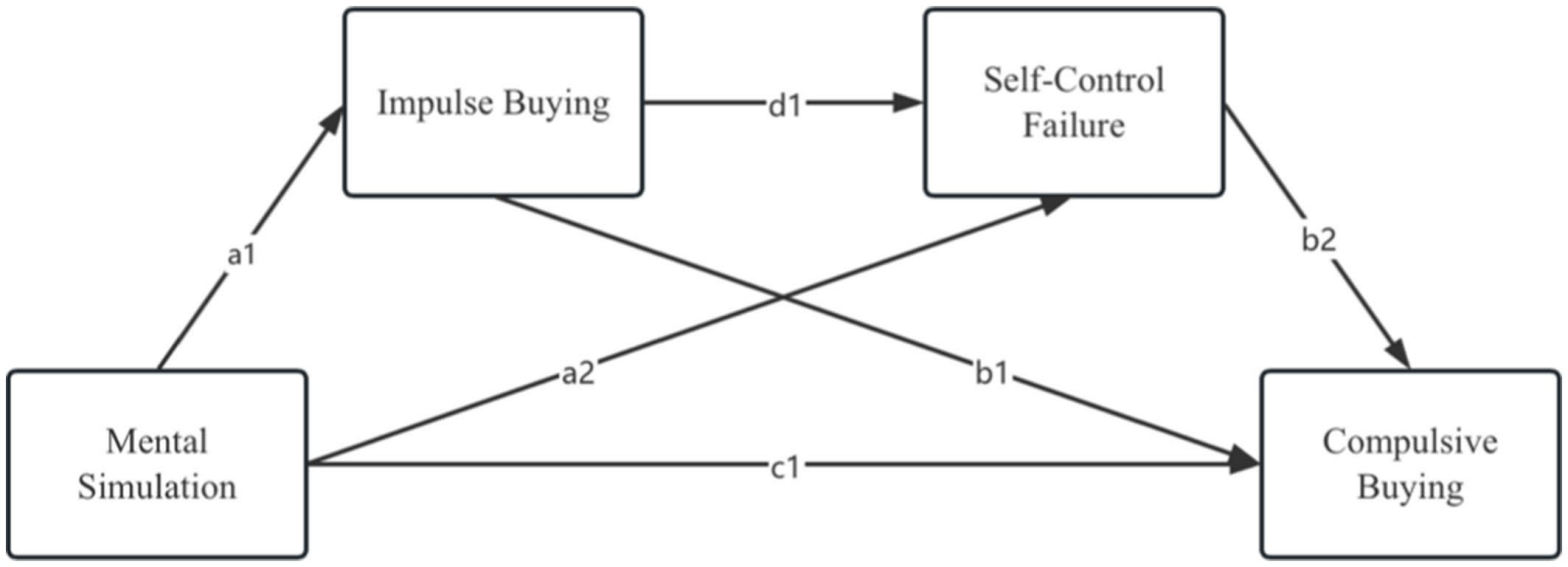
Conceptual framework. H1: c1; H2: a1*b1; H3: a2*b2; H4: a1*d1*b2.

Considering the above, the present study postulates that impulse buying and self-control failure exert mediation effects on the relationships between the two types of mental simulation and compulsive buying. Additionally, consistent with the above statements, the overall findings of existing research also support the significant association between impulse buying behaviors and loss of self-control. Against this background, the current study adopts an integrated multiple mediation model, as presented by Hayes (2013), which is conducive to generating further insights into the links between the two types of mental simulation and compulsive buying. Moreover, an integrated multiple mediation model is effective in improving the compatibility and accuracy of the established conceptual framework by means of parallel, sequential, or mixed comparisons between two mediators (Hayes, 2013). To the best of our knowledge, the serial multiple mediation effects of impulse buying and self-control failure on the relationship between mental simulation and compulsive buying remain empirically unexplored to date. It is therefore imperative to investigate the underlying mechanisms between these two mediators. The aforementioned discussion supports the next two hypotheses and the conceptual framework is illustrated in Figure 1.

*H4a*: Impulse buying and self-control failure serially mediate the link between outcome simulation and compulsive buying.

*H4b*: Impulse buying and self-control failure serially mediate the link between process simulation and compulsive buying.

## 3 Methods

### 3.1 Data collection

We conducted a web-based survey using established scales from existing literature and recruited 220 respondents through the Nown Survey randomly. A total of 220 responses were collected, of these, 18 incomplete responses were eliminated. Thus, 202 responses were used as the final sample consisted of 45.54% male respondents and 54.46% female respondents. For the bracket of age, the majority of respondents are between 20 and 24 years old (36.63%), followed by the age of 25–29 (27.72%), the age of 30–34 (11.39%), the age of 35–39 (10.89%), below 20 years old (7.92%), and above 40 years old (5.45%), respectively. For the education background, about 78.22% of total respondents have achieved a bachelor’s degree, especially, those who with a master’s degree account for 26.24% of total respondents. In addition, the monthly income of majority is between 1,000 K and 2,000 K (55.45%), followed by the 500–1,000 K income bracket (17.33%), below 500 K (14.85%), and above 2,000 K (12.37%), respectively. For the bracket with monthly incomes between 1,000 K and 1,500 K (27.23%) and 1,500 K to 2,000 K (28.22%), their higher income levels typically make them more inclined to consume luxury goods. These consumers may allocate a portion of their disposable income to purchase fashionable clothing, cosmetics, and other affordable luxury goods to enhance their quality of life and social image. The bracket with incomes between 500 K and 1,000 K (17.33%), although relatively lower in income, may still opt for affordable luxury products or smaller luxury items (wallets, hats, scarves, etc.), especially during sales or promotional periods, as they might hope to achieve greater brand value within a limited budget. Additionally, high-income bracket (1,500 K and above) are usually willing to invest in the latest smartphones, tablets, and other electronic devices. Middle-income consumers (1,000 K to 1,500 K) are also likely to engage in electronic product consumption. For them, the quality and functionality of electronic products are primary considerations. More detailed demographic characteristics of respondents appear in Table 1.

**Table 1:** Demographic characteristics.

Measure	Items	Frequency	Percentage (%)
Gender	Male	92	45.54
	Female	110	54.46
Age	< 20 years old	16	7.92
	20–24 years old	74	36.63
	25–29 years old	56	27.72
	30–34 years old	23	11.39
	35–39 years old	22	10.89
	> = 40 years old	11	5.45
Highest education	High school or below	19	9.40
	2-year college	25	12.38
	Bachelors	105	51.98
	Masters or higher	53	26.24
Income (KRW/Month)	< 500 K	30	14.85
	500–1,000 K	35	17.33
	1,000–1,500 K	55	27.23
	1,500–2,000 K	57	28.22
	> 2,000 K	25	12.37

*N = 202.*

### 3.2 Measures

The measure items of impulse buying are adapted from an established scale by Rook and Fisher (1995). For instance, “I often buy things spontaneously,” “I often buy things without thinking,” and so forth. The compulsive buying scale is modified from a study by Faber and O’Guinn (1992). For example, “I often buy things even though I could not afford them,” “Others would feel horrified if they knew of my spending habits,” and so forth. Lastly, the 4-item scale of self-control is revised from a self-control scale presented by Tangney et al. (2004). For instance, “I do certain things that are bad for me, if they are fun,” “Pleasure and fun sometimes keep me from getting work done,” and so forth. Moreover, all scales utilized in this study employ a 5-point Likert scale ranging from “1 = strongly disagree” to “5 = strongly agree.” Table 2 reports all measure items employed in the current study.

**Table 2:** Measurement items.

Construct	Description	Sources
Impulse Buying	I often buy things spontaneously.	Rook and Fisher (1995)
	I often buy things without thinking.	
	I sometimes feel like buying things on the spur-of-the-moment.	
Compulsive buying	I often buy things even though I could not afford them.	Faber and O’Guinn (1992)
	Others would feel horrified if they knew of my spending habits.	
	If I have any money left at the end of the pay period, I just have to spend it.	
	I often buy something in order to make myself feel better.	
Self-control	I do certain things that are bad for me, if they are fun.	Tangney et al. (2004)
	Pleasure and fun sometimes keep me from getting work done.	
	I sometimes cannot stop myself from doing something, even if I know it is wrong.	
	I often act without thinking through all the alternatives.	

### 3.3 Manipulations and check

Informed by existing literature, the manipulation of mental simulation is usually conducted by either scenarios or images. Following the verified scenario created by Zhao et al. (2011), this study employs analogous scenarios to prime participants’ mental simulation. First, in advance of the priming stage, participants are provided with a scenario that “if you went to a Louis Vuitton store to buy a wallet that you were fancying before, however, you stumbled upon another new collection of the wallet which is more expensive and fascinating.” Participants are, then, asked to record their purchase intentions on the latest wallet ranging from “1 = a weak purchase intention” to “5 = a strong purchase intention.” Second, to prime outcome simulation, participants are informed by a same scenario mentioned before. However, before recording their purchase intention scores, participants are asked to concretely imagine several affective benefits, such as the envy from others, positive feelings (e.g., pleasure and excitement) derived from the new wallet, and so forth. In contrast, to prime participants’ process simulation, a similar scenario is presented. That is, “if you went to an Apple store to purchase an iPod, however, you met a new iPhone promotion event occasionally.” Then participants are asked to make a record of their purchase intentions for the new iPhone; then they, in turn, imagine some cognitive benefits when using the new iPhone, such as favorable running speed of the new iPhone, convenience derived from new functions. Finally, participants are asked to record their purchase intentions once again.

We conducted a paired-samples *T*-test to check whether the manipulation is statistically successful. In line with our results, for the manipulation of outcome simulation, the purchase intention_before_ (*M* = 2.32, SD = 0.886) is significantly distinct from the purchase intention_after_ (*M* = 2.94, SD = 1.023, *t* = −9.007, *p* < 0.001). Likewise, for the manipulation of process simulation, a significant distinction between the purchase intention_before_ (*M* = 2.17, SD = 0.557) and the purchase intention_after_ (*M* = 2.65, SD = 0.661) is also reported (*t* = −11.485, *p* < 0.001). According to the foregoing statement, the manipulation can be considered as being successful. Table 3 describes the results of manipulation check.

**Table 3:** Manipulation check results.

	*N*	*M*	SD	*t*	*p*
OS-PI_before_	202	2.32	0.886	−9.007	0.000^***^
OS-PI_after_	202	2.94	1.023		
PS-PI_before_	202	2.17	0.557	−11.485	0.000^***^
PS-PI_after_	202	2.65	0.661		

OS, Outcome Simulation; PS, Process Simulation; PI, Purchase Intention.

^***^, Correlation is significant at the 0.001 level (2-tailed).

### 3.4 Results

#### 3.4.1 Reliability and validity

To confirm the robustness of the scales, a confirmatory factor analysis (CFA), a correlation analysis, and a reliability analysis are conducted. First, the Cronbach’s *α* yielded from a reliability analysis for impulse buying is 0.856, compulsive buying is 0.811, self-control is 0.802, outcome simulation is 0.737, and process simulation is 0.741, all coefficients exceed the recommended value of 0.70 (Kline, 2011), thus, an acceptable reliability is confirmed. Second, as shown in Table 4, the diagonal elements of correlation matrix (i.e., square roots of AVE) is over the off-diagonal elements (i.e., correlation coefficients of variables), hence, showing a satisfactory discriminant validity. Third, the standardized factor loadings ranging from 0.728 to 0.966 are all greater than the suggested cut-off value of 0.70 (Fornell and Larcker, 1981), the values of CR are all greater than the recommended value of 0.70 (Hair et al., 2006), the values of AVE for all constructs all exceed the threshold of 0.50 (Fornell and Larcker, 1981), as reported in Table 5. Hence, a favorable divergent validity is established.

**Table 4:** Descriptive statistics and correlation matrix of all constructs.

Construct	M	SD	1	2	3	4	5
1 Impulse buying	3.368	1.067	0.881				
2 Compulsive buying	3.205	0.922	0.570^**^	0.799			
3 Self-control	3.253	0.806	0.453^**^	0.467^**^	0.792		
4 Outcome simulation	2.626	0.823	0.538^**^	0.455^**^	0.433^**^	0.851	
5 Process simulation	2.411	0.532	0.435^**^	0.478^**^	0.342^**^	0.545^**^	0.855

Diagonal elements of correlation matrix are square roots of AVE.

^**^, Correlation is significant at the 0.01 level (2-tailed).

**Table 5:** Construct reliability and validity.

Construct	Indicators	Standardized factor loadings	CR	AVE	CA
Impulse buying	IB1	0.846	0.912	0.776	0.856
	IB2	0.893			
	IB3	0.903			
Compulsive buying	CB1	0.781	0.876	0.639	0.811
	CB2	0.809			
	CB3	0.858			
	CB4	0.745			
Self-control	SC1	0.805	0.871	0.627	0.802
	SC2	0.811			
	SC3	0.788			
	SC4	0.764			
Outcome simulation	OS1	0.751	0.839	0.725	0.737
	OS2	0.941			
Process simulation	PS1	0.728	0.842	0.731	0.741
	PS2	0.966			

CR, Composite Reliability; AVE, Average Variance Extracted; CA, Cronbach’s.

#### 3.4.2 Hypotheses testing

To test the established hypotheses, two sub-models are built and investigated using the PROCESS for SPSS (Model 6). In the model 1, in terms of the relationship between impulse buying and self-control failure, the results show that there exists a significant association between these two constructs (*b* = 0.421, *p* < 0.001). Regarding the relationship between self-control failure and compulsive buying, the results here also indicate that self-control failure is a significant predictor of compulsive buying (*b* = 0.335, *p* < 0.001). As such, the significant correlation between impulse buying and compulsive buying is confirmed (*b* = 0.469, *p* < 0.001). Moreover, the results exhibit that outcome simulation is a significant antecedent to impulse buying (*b* = 0.763, *p* < 0.001) and to self-control failure (*b* = 0.135, *p* < 0.05). Nonetheless, the significant relation between outcome simulation and compulsive buying is not found (*b* = 0.097, *p* > 0.05).

**Figure 2:**
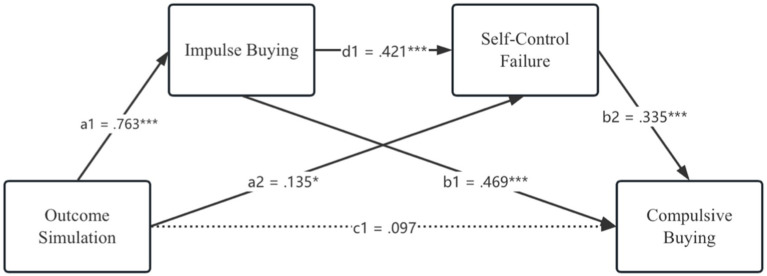
Research model testing results 1.

With regard to mediation effects, the direct effect, that is, the negative association between outcome simulation and compulsive buying is not supported (*b* = 0.097, CI [−0.016, 0.209]), therefore, H1a is rejected. Regarding the mediation effect of impulse buying, the results show that impulse buying significantly mediates the link between outcome simulation and compulsive buying (*b* = 0.358, CI [0.250, 0.479]), which supports H2a accordingly. In terms of the mediating role of self-control failure, the results indicate that self-control failure significantly mediates the relationship between outcome simulation and compulsive buying (*b* = 0.045, CI [0.002, 0.101]), lending credence to H3a. Likewise, it is construed that the association between outcome simulation and compulsive buying is sequentially mediated by individuals’ impulse buying behaviors and loss of self-control (*b* = 0.108, CI [0.057, 0.180]), hence, H4a is accepted. Moreover, in terms of the whole multiple mediation model, both indirect and total effects are significant, however, the direct effect is insignificant, which indicates the presence of full mediation. Table 6 and Figure 2 report more detailed results.

**Table 6:** Summary of multiple mediation model 1 testing results.

Construct effects	*b*	95% CI		*t*-values
		Lower	Upper	
OS ➔ IB	0.763^***^	0.617	0.909	10.282
OS ➔ SC	0.135^*^	0.006	0.264	2.067
OS ➔ CB	0.097	−0.016	0.209	1.689
IB ➔ SC	0.421^***^	0.322	0.520	8.355
SC ➔ CB	0.335^***^	0.214	0.456	5.461
IB ➔ CB	0.469^***^	0.370	0.569	9.265

Mediation effects	*b*	95% CI		Relative effects
		Lower	Upper	
Direct effect (H1a)	0.097	−0.016	0.209	
OS ➔ IB ➔ CB (H2a)	0.358	0.250	0.479	58.88%
OS ➔ SC ➔ CB (H3a)	0.045	0.002	0.101	7.40%
OS ➔ IB ➔ SC ➔ CB (H4a)	0.108	0.057	0.180	17.76%
Total effect	0.608^***^	0.476	0.739	

IB, Impulse Buying; SC, Self-Control; CB, Compulsive Buying; OS, Outcome Simulation.

^*^p < 0.05, ^**^p < 0.01, ^***^p < 0.001.

In the model 2, with regard to the relationship between impulse buying and self-control failure, impulse buying is positively associated with self-control failure (*b* = 0.456, *p* < 0.001). Likewise, the association between self-control failure and compulsive buying is significantly confirmed (*b* = 0.333, *p* < 0.001). Results also show that impulse buying is positively related to compulsive buying (*b* = 0.459, *p* < 0.001). In addition, as expected, process simulation is significantly associated with compulsive buying (*b* = 0.248, *p* < 0.01). In terms of the relationship between process simulation and impulse buying, process simulation is significantly associated with impulse buying, however, the hypothesized negative relation between these two variables is not confirmed (*b* = 0.899, *p* < 0.001). Furthermore, the results indicate that the significant relationship between process simulation and self-control failure is not found (*b* = 0.119, *p* > 0.05).

**Figure 3:**
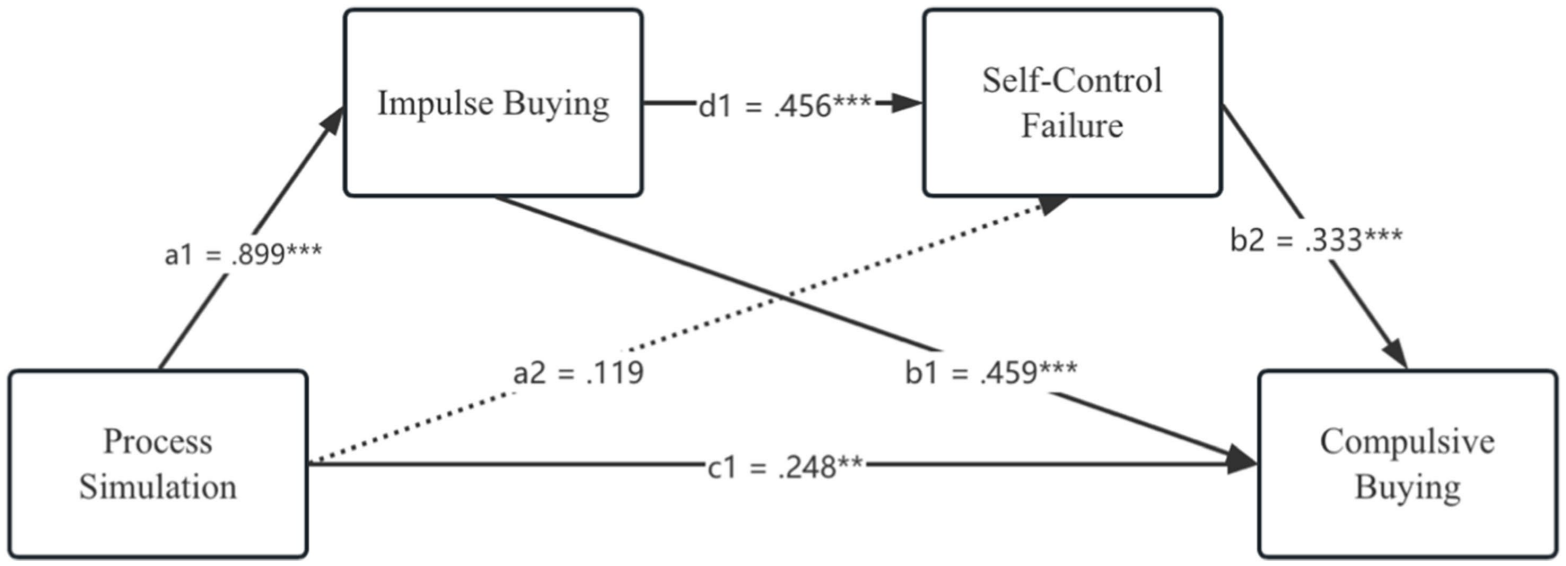
Research model testing results 2.

With regard to the positive relationship between process simulation and compulsive buying, the results manifest that there exists a significant association between these two constructs (*b* = 0.248, CI [0.094, 0.402]), hence, H1b is supported. The results also elucidate that individuals’ impulsive purchasing behaviors mediate the relationship between process simulation and their compulsive buying behaviors (*b* = 0.412, CI [0.270, 0.566]), H2b is accepted accordingly. Nevertheless, in terms of H3b, the significant mediation effect of self-control failure on the link between process simulation and compulsive buying is not found (*b* = 0.040, CI [−0.031, 0.131]), in response, H3b is rejected. Additionally, the results indicate that the pathway of “process simulation → impulse buying →self-control failure → compulsive buying” is also significant, that is, impulse buying and self-control failure serially mediate the relation between process simulation and compulsive buying (*b* = 0.136, CI [0.075, 0.218]), thus, H4b is supported. Furthermore, with regard to the whole multiple mediation model, the significant direct, indirect, and total effects jointly indicate the presence of partial mediation. More detailed results appear in Table 7 and Figure 3.

**Table 7:** Summary of multiple mediation model 2 testing results.

Construct effects	*b*	95% CI		*t*-values
		Lower	Upper	
PS ➔ IB	0.899^***^	0.649	1.149	7.085
PS ➔ SC	0.119	−0.063	0.300	1.289
PS ➔ CB	0.248^**^	0.094	0.402	3.180
IB ➔ SC	0.456^***^	0.365	0.546	9.934
SC ➔ CB	0.333^***^	0.214	0.451	5.556
IB ➔ CB	0.459^***^	0.365	0.552	9.684

Mediation effects	*b*	95% CI		Relative effects
		Lower	Upper	
Direct effect (H1b)	0.248^**^	0.094	0.402	29.67%
PS ➔ IB ➔ CB (H2b)	0.412	0.270	0.566	49.28%
PS ➔ SC ➔ CB (H3b)	0.040	−0.031	0.131	
PS ➔ IB ➔ SC ➔ CB (H4b)	0.136	0.075	0.218	16.27%
Total effect	0.836^***^	0.625	1.048	

IB, Impulse Buying; SC, Self-Control; CB, Compulsive Buying; PS, Process Simulation.

^*^p < 0.05, ^**^p < 0.01, ^***^p < 0.001.

## 4 General discussion

### 4.1 Conclusion

The results show that, first, outcome simulation is significantly related to both impulse buying and self-control failure, while no significant relationship was found between outcome simulation and compulsive buying. Process simulation is significantly associated with both impulse buying and compulsive buying. A plausible explanation is that process simulation may lead to a dual focus on both the mean (i.e., step-by-step process) and the end benefit (i.e., desired outcome; Thompson et al., 2009). To restate, the focus on means and end benefits may remain simultaneously prominent; thus, it is construed that process simulation with a dual focus is effective in evoking both impulse and compulsive buying behaviors (Thompson et al., 2009). Second, the significant relationship between impulse buying and compulsive buying was empirically confirmed. Simultaneously, a mediating effect of self-control failure on the relationship between impulse buying and compulsive buying was also found. Despite the significant association between impulse buying and compulsive buying, the distinctions between these two constructs remain salient. A primary differentiator between impulse buying and compulsive buying is the individual’s perception of affect (Flight et al., 2012). In other words, impulse buying behavior is highly related to positive feelings, whereas compulsive buying behavior is closely linked to negative emotional states (Flight et al., 2012). Lastly, the results show that the serial multiple mediation effects of impulse buying and self-control failure on the relationship between the two types of mental simulation and compulsive buying are significant. More specifically, it is demonstrated that impulse buying and self-control failure separately and sequentially mediate the relationship between outcome simulation and compulsive buying. However, no mediating effect of self-control failure was found on the relationship between process simulation and compulsive.

### 4.2 Theoretical and managerial implications

#### 4.2.1 Theoretical implications

Several implications related to the results of this study are presented accordingly. Regarding theoretical implications, first, this study advances existing knowledge on the link between impulse buying and compulsive buying by highlighting the mediation effect of self-control failure on the relationship between impulse buying and compulsive buying. Simultaneously, this research emphasizes how self-control failure serves as a pivotal mediator influencing consumers’ impulse buying and compulsive buying behaviors. These findings support the relevant literature on self-control theory, indicating that when consumers face temptations (promotions, advertisements, etc.), the abilities of self-control directly affect their decision making (Baumeister, 2002). Second, the current study appears to be among the first to examine the serial mediation effects of impulse buying and self-control failure on the relationship between mental simulation and compulsive buying. And this study clearly distinguishes between impulse buying (i.e., spontaneous purchase urges) and compulsive buying (i.e., uncontrollable purchase urges), which helps construct a more detailed consumer behavior model. This work not only enriches the theoretical foundation of consumer behavior studies but also provides a framework for future empirical research. Third, this research explores how emotions (desire, anxiety, etc.) influence consumers’ impulse buying, and how these emotions lead to compulsive buying behaviors following self-control failure. The interaction between emotions and cognitions provides a new perspective for understanding consumers’ purchasing motivations (Nikolinakou et al., 2024; Japutra et al., 2025). Lastly, our findings contribute to the emerging research stream on mental simulation by illustrating that two types of mental simulation differ in driving consumers’ impulse and compulsive buying behaviors. The results suggest that consumers’ decision making is profoundly influenced by situational and psychological cues. This insight advances the understanding of different aspects of consumer behavior and may guide future research in exploring emerging consumption patterns.

#### 4.2.2 Managerial implications

In terms of managerial implications, compulsive buying is considered as problematic purchase behavior in a broader sense, as it can be detrimental to a brand’s reputation in the long run (Billieux et al., 2008; Japutra et al., 2019). Simultaneously, based on the overall findings of this study, outcome simulation is associated only with impulse buying, while process simulation is positively linked to both impulse buying and compulsive buying. In this regard, these findings can help top managers to establish effective strategies in terms of the adoption of mental simulation in advertisements. In other words, when inducing consumers’ purchase behaviors, marketers should employ an outcome simulation strategy, rather than a process simulation approach that may yield problematic purchase behavior (i.e., compulsive buying). For instance, Lemon and Verhoef (2016) points out that successful customer relationship management (CRM) is not just a technical issue, it is also about how to utilize data to enhance customer experience. An efficient CRM system can collect consumer data from multiple channels (e-commerce platforms, social media, offline stores, etc.), including purchase history, browsing records, and feedback. By integrating the information, a comprehensive consumer profile can be built to help businesses identify which customers are prone to compulsive buying. Furthermore, setting up feedback channels within the CRM system allows consumers to share their experiences and feelings promptly after shopping. This feedback can be used to adjust products and services, particularly for those showing tendencies of compulsive buying, helping businesses understand and meet their needs.

Further, the results show that the mediating roles of impulse buying and self-control failure on the relationship between the two types of mental simulation and compulsive buying are divergent. That is, impulse buying and self-control failure fully mediate the relationship between outcome simulation and compulsive buying; in contrast, impulse buying and self-control failure exert only partial mediation effects on the relationship between process simulation and compulsive buying. To this end, the current study suggests that marketing practitioners should incorporate these findings into more marketing activities, such as new product development, segmentation structure establishment, and advertising. The marketing practitioners can effectively utilize mental simulation in advertisements, particularly outcome simulation, to promote impulse buying rather than compulsive buying. That is to guide consumers’ decisions by showcasing positive outcomes in the advertisements. Sweeney and Soutar (2001) indicates that consumers’ perceived value significantly influences their purchasing decisions. By optimizing advertising design and strengthening outcome simulation, it can enhance consumers’ perception of product value, thereby driving sales. For example, sharing positive experiences from customers after using the product can illustrate the specific benefits. This approach can resonate with consumers, making it easier for them to envision a positive future after purchasing the product.

### 4.3 Limitations and future research

There are certain limitations to this study that should be noted. First, the sample size of the current study was comparatively small. To increase both the external validity of the measurement and the generalizability of the results, future research should recruit more respondents to optimize data collection. Second, the study was limited to a singular research method. Future studies could employ more diverse research approaches, such as an experimental approach and a longitudinal approach. Third, this study focuses on the effects of different types of mental simulation on impulse buying, self-control failure, and compulsive buying. In other words, this academic work aims at exploring the consequences of mental simulation. In fact, previous research has also discussed various antecedents of mental simulation (Jin, 2016), and these findings are sufficiently noteworthy and meaningful. It is believed that incorporating the antecedents of mental simulation into future research models will yield more interesting discoveries. At the same time, this study, like most existing research, views self-control as a holistic concept rather than a multidimensional one. If future studies could explore the components of self-control, it is likely that more significant findings will emerge. Lastly, this study places greater focus on the positive aspects of affect in consumers’ impulse buying behaviors. However, drawing upon past research, high levels of impulsive purchase behaviors can be considered as a self-destructive buying behavior, which may be induced by dispositional negative affect, such as a lack of self-esteem (Verplanken et al., 2005). Likewise, consumers’ impulse buying may be motivated by their attempts to mitigate negative emotional states, which is concordant with the theory of self-gifting (Mick and Demoss, 1990). In response, researchers could make more efforts to explore the association between individuals’ negative emotional responses and their impulse buying behaviors in future research.

## Data Availability

The raw data supporting the conclusions of this article will be made available by the authors, without undue reservation.
